# Long-Term Excessive Dietary Phosphate Intake Increases Arterial Blood Pressure, Activates the Renin–Angiotensin–Aldosterone System, and Stimulates Sympathetic Tone in Mice

**DOI:** 10.3390/biomedicines10102510

**Published:** 2022-10-07

**Authors:** Nejla Latic, Mirko Peitzsch, Ana Zupcic, Jens Pietzsch, Reinhold G. Erben

**Affiliations:** 1 Department of Biomedical Sciences, University of Veterinary Medicine, 1210 Vienna, Austria; 2 Institute of Clinical Chemistry and Laboratory Medicine, University Hospital Carl Gustav Carus, 01307 Dresden, Germany; 3 Helmholtz-Zentrum Dresden-Rossendorf, Institute of Radiopharmaceutical Cancer Research, Department of Radiopharmaceutical and Chemical Biology, 01328 Dresden, Germany; 4 School of Science, Faculty of Chemistry and Food Chemistry, Technische Universität Dresden, 01062 Dresden, Germany

**Keywords:** hypertension, left ventricular hypertrophy, renin–angiotensin–aldosterone system, cardiovascular disease, catecholamines, liquid chromatography–tandem mass spectrometry

## Abstract

Increased dietary phosphate intake has been associated with severity of coronary artery disease, increased carotid intima–media thickness, left ventricular hypertrophy (LVH), and increased cardiovascular mortality and morbidity in individuals with normal renal function as well as in patients suffering from chronic kidney disease. However, the underlying mechanisms are still unclear. To further elucidate the cardiovascular sequelae of long-term elevated phosphate intake, we maintained male C57BL/6 mice on a calcium, phosphate, and lactose-enriched diet (CPD, 2% Ca, 1.25% P, 20% lactose) after weaning them for 14 months and compared them with age-matched male mice fed a normal mouse diet (ND, 1.0% Ca, 0.7% P). Notably, the CPD has a balanced calcium/phosphate ratio, allowing the effects of elevated dietary phosphate intake largely independent of changes in parathyroid hormone (PTH) to be investigated. In agreement with the rationale of this experiment, mice maintained on CPD for 14 months were characterized by unchanged serum PTH but showed elevated concentrations of circulating intact fibroblast growth factor-23 (FGF23) compared with mice on ND. Cardiovascular phenotyping did not provide evidence for LVH, as evidenced by unchanged LV chamber size, normal cardiomyocyte area, lack of fibrosis, and unchanged molecular markers of hypertrophy (*Bnp*) between the two groups. However, intra-arterial catheterization revealed increases in systolic pressure, mean arterial pressure, and pulse pressure in mice fed the CPD. Interestingly, chronically elevated dietary phosphate intake stimulated the renin–angiotensin–aldosterone system (RAAS) as evidenced by increased urinary aldosterone in animals fed the CPD, relative to the ND controls. Furthermore, the catecholamines epinephrine, norepinephrine, and dopamine as well as the catecholamine metabolites metanephrine. normetanephrine and methoxytyramine as measured by mass spectrometry were elevated in the urine of mice on CPD, relative to mice on the ND. These changes were partially reversed by switching 14-month-old mice on CPD back to ND for 2 weeks. In conclusion, our data suggest that excess dietary phosphate induces a rise in blood pressure independent of secondary hyperparathyroidism, and that this effect may be mediated through activation of the RAAS and stimulation of the sympathetic tone.

## 1. Introduction

Phosphate is part of the fundamental chemical necessary for cellular structure, signaling and energy production, making it essential for various biological processes. Phosphate metabolism is mainly regulated by the vitamin D hormone (1,25(OH)_2_D_3_) and by two major phosphaturic hormones, fibroblast growth factor 23 (FGF23) and parathyroid hormone (PTH). FGF23 and PTH act on proximal tubules in the kidney to increase renal phosphate excretion by inhibiting reabsorption of filtered phosphate. Therefore, FGF23 and PTH lower phosphate levels in the blood. 1,25(OH)_2_D_3_ on the other hand stimulates phosphate absorption from the intestine, thereby increasing serum phosphate levels [1].

Despite its importance for various processes in the body, the accumulation of phosphate in the blood, hyperphosphatemia, can have deleterious effects. Epidemiological and observational studies have reported an association between increased serum phosphate levels and dietary phosphate load with left ventricular hypertrophy (LVH), cardiac calcification, as well as morbidity and mortality in patients suffering from chronic kidney disease (CKD) [2,3,4,5]. Cardiovascular events are the most frequent cause of death in CKD patients [6]. As the glomerular filtration rate decreases in CKD, the kidney is unable to adequately excrete phosphate, leading to hyperphosphatemia. Hyperphosphatemia has been identified as an independent risk factor for accelerated cardiovascular disease (CVD) development in these patients, but the exact mechanisms have not yet been elucidated [7]. Interestingly, high serum phosphate levels are also associated with an increased risk for CVD in adults with no history of CVD or CKD [8,9,10].

High dietary phosphate may also be linked to hypertension. Hypertension is a major health burden affecting about 1.3 billion people worldwide [11]. The existing epidemiological data on the effect of dietary phosphate on hypertension are inconsistent. While some studies have found a clear association between phosphate intake and hemodynamic parameters, others have not observed changes in blood pressure that were attributable to a high dietary phosphate load [10,12,13]. A recent intervention study shed additional light on this issue. Mohammad and coworkers examined the effects of a high phosphate diet in healthy subjects with normal renal function, and found that a 6-week dietary phosphate load induced small, but significant increases in systolic and diastolic blood pressure that were accompanied by activation of the sympathetic tone, relative to subjects receiving the low phosphate diet [10].

It still remains an open question whether the untoward effects of excess dietary phosphate on the cardiovascular system are direct or indirect. Experimental studies have attempted to clarify the mechanisms involved. Studies supporting a direct role of phosphate postulate that hyperphosphatemia leads to arterial calcification by directly stimulating the differentiation of vascular smooth muscle cells into osteoblast-like cells [14,15,16]. Furthermore, a study in rat aortas demonstrated that an increase in extracellular phosphate is followed by an increase in oxidative stress, a decrease in nitric oxide production, and inhibitory phosphorylation of endothelial nitric oxide synthase (eNOS) [17]. On the other hand, phosphate may act indirectly by inducing changes in the endocrine regulators of phosphate homeostasis, FGF23, PTH and 1,25(OH)_2_D_3_. An increase in serum phosphate stimulates FGF23 and PTH secretion, while at the same time inhibiting production of 1,25(OH)_2_D_3_. In this context, it is established that FGF23 causes LVH via FGF receptor 4-dependent activation of the calcineurin-NFAT signaling pathway [17]. PTH may cause hypertension by its direct effects on arteries and myocytes to promote arterial stiffness and LVH [18]. Indeed, Bozic and coworkers found an increase in blood pressure and LVH in normotensive and spontaneously hypertensive rats with normal kidney function that was driven by an increase in PTH [19].

It is clear that elucidation of the mechanisms underlying the potential hypertensive and hypertrophy-promoting effects of a high phosphate intake may have major implications for the prevention of CVD and its sequelae in humans. Previous experimental studies in rodents mostly used a phosphate-enriched diet to investigate the effects of an increased dietary phosphate intake on the cardiovascular system or the kidney [19,20,21,22]. However, an isolated increase in dietary phosphate leads to secondary hyperparathyroidism, making it difficult to dissect the effects of phosphate from that of increased PTH on cardiovascular endpoints. In this study, we sought to further explore the cardiovascular sequelae of long-term elevated phosphate intake in aged mice, largely independent of changes in PTH. To this end, we maintained male C57BL/6 mice on a calcium, phosphate, and lactose-enriched diet (CPD) with a balanced calcium/phosphate ratio for 14 months and compared them with age-matched mice fed a normal mouse diet. We found that 14-month-old mice on a CPD were characterized by hypertension and increased arterial stiffness, and that these changes were associated with increased urinary aldosterone excretion and augmented sympathetic tone.

## 2. Material and Methods

### 2.1. Animals

The study was undertaken in accordance with prevailing EU and national guidelines for animal care and welfare and in compliance with ARRIVE guidelines. All animal procedures were approved by the Ethics and Animal Welfare Committee of the University of Veterinary Medicine Vienna, Austria, and by the Austrian Federal Ministry of Education, Science and Research (permit number BMWFW-68.205/0188-WF/V/3b/2017).

All experiments were performed using 12–14-month-old male C57BL/6 mice. Animals were kept on 24 °C with a 12/12-h light/dark cycle and were housed in stable groups of 2–5 mice from the time of weaning. Starting from weaning, the mice were fed either a normal mouse diet (V1124-000, Sniff, Soest, Germany) containing 1.0% calcium, 0.7% phosphorus, and 1000 IU vitamin D/kg, or a CPD containing 2% Ca, 1.25% P, and 20% lactose. The Ca/P ratio of both diets was similar (1.43 for ND and 1.60 for CPD), and both diets had the same energy content (14 MJ/kg) and the same nutrient distribution (carbohydrates, protein, fat). All animals had access to food and tap water ad libitum. Before necropsy, urine was collected overnight in metabolic cages. At necropsy, the mice were exsanguinated from the abdominal vena cava under general anesthesia with ketamine/medetomidine (100/0.25 mg/kg i.p.) for serum collection. Necropsies were performed between 9 a.m. and 3 p.m. for all mice. Some mice maintained on a CPD until 12–14 months of age were switched to ND for two weeks to assess the effect of the diet switch on urinary excretion of catecholamines. Investigators were not blinded to the group allocation of the animals.

### 2.2. Biochemical Analysis

Serum and urinary phosphate, calcium, sodium, and creatinine were measured using a Cobas c111 analyzer (Roche, Mannheim, Germany). Serum intact FGF23 (Kainos, Tokyo, Japan), serum and urinary aldosterone (NovaTec Immundiagnostica, Dietzenbach, Germany), serum intact PTH (Immutopics Inc, San Clemente, CA, USA), and serum renin (Abbexa Ltd., Cambridge, UK) were detected using commercially available ELISA kits according to the manufacturer’s instructions. Serum was extracted with diethylether and re-suspended in steroid-free serum (DRG Diagnostics, Marburg, Germany) for the aldosterone ELISA.

### 2.3. RNA Isolation and Quantitative RT-PCR

Total RNA isolation and quantitative RT-PCR analysis were performed as described previously [23]. All samples were measured in triplicate and normalized to two housekeeping genes (*Dpm1 and Txnl4a*). The qPCR results were obtained and evaluated with the software qPCRsoft 4.1 (V4.1.3.0, Analytik Jena, Jena, Germany), and then analyzed using the standard delta delta Cq method.

### 2.4. Echocardiography

Echocardiography was performed one day before the necropsies using a 14MHz linear transducer (Siemens Accuson s2000, Munich, Germany) for evaluation of cardiac function. Mice were under 1% isoflurane anesthesia with a stable body temperature of 37 °C. M-mode in short-axis at the level of the papillary muscles was used to evaluate LV thickness, fractional shortening, and internal diameters in systole and diastole. At least four cardiac cycles were analyzed for each parameter.

### 2.5. Central Arterial and Cardiac Pressure Measurements and Augmentation Index

Central arterial pressure was measured by inserting a micro-tip catheter (1.4 Millar Instruments, Houston, TX, USA) into the ascending aorta via the right carotid artery under 1.5% isoflurane anesthesia. The catheter was then further advanced into the left ventricle to obtain cardiac pressure parameters. Traces were recorded for at least three minutes and analyzed via LabChart7 software (ADInstruments, Dunedin, New Zealand). The aortic augmentation index was identified from the late systolic portion of the arterial pressure wave as described previously [24]. The augmentation index was defined as the height from the augmentation point to the systolic peak of the pressure wave divided by the pulse pressure, and was expressed as a percentage.

### 2.6. Histological Evaluation

Hearts were fixed in 4% paraformaldehyde, and then paraffin-embedded and cut into 5 µm sections. Cardiac sections were stained with FITC-labeled wheat germ agglutinin for the analysis of cardiomyocyte size. At least 10 random areas of the heart were measured and only cardiomyocytes with well-defined borders and visible nuclei were used. Images were obtained by the Zeiss LSM 880 Airyscan confocal microscope (Zeiss, Oberkochen, Germany) and analyzed using Image J software. Fibrotic tissue was visualized by staining with picrosirius red according to a standard protocol. Total collagen was quantified using ImageJ software and was expressed as the ratio of collagen-stained area to total muscle area of the left ventricle and septum. All histological images were analyzed by two independent investigators in a blinded manner.

### 2.7. Urinary Catecholamine Measurement

Mice were placed in individual cages for spontaneous urine collection. At least 150 ul was collected and used for further analysis. The urinary concentration of the catecholamines and catecholamine metabolites epinephrine, norepinephrine, dopamine, normetanephrine, metanephrine, and methoxytyramine were measured using liquid chromatography–tandem mass spectrometry (LC–MS/MS) as described previously [25].

### 2.8. Statistical Analysis

Statistical planning of the experiment was based on a minimum group size of 10 mice per diet, and was performed based on the variance of the main target variable, mean arterial blood pressure. Since the mice were bred in our own animal facility, and the experiments were built up using cohorts of mice, the number of mice per diet group ranged between 12–16. A different set of mice (*n* = 6 per group) was used for measurement of urinary catecholamines, and for the diet switch experiment. One animal was excluded from the analysis of urinary catecholamines because this animal, for unknown reasons, showed about 4-fold lower values compared with the rest of the group for all metabolites measured. Some values for specific measurements were excluded from the analysis if the data acquired were out of the defined range for a specific measurement.

Statistical analysis was performed using Graph Pad Prism 9 (GraphPad Software, San Diego, CA, USA). The data were analyzed by two-sided Mann–Whitney U-test for comparison of mice maintained on ND or CPD, or by Pearson’s correlation analysis, followed by linear regression analysis. Urinary levels of catecholamines and their metabolites before and after switching the diet were analyzed using the Wilcoxon matched-pairs test. *p* values of less than 0.05 were considered significant. Data are presented as scatter dot plots with bars depicting means ± SEM.

## 3. Results

### 3.1. Mice Maintained on CPD for 14 Months have Increased Levels of FGF23, but Normal Kidney Function

**Figure 1:**
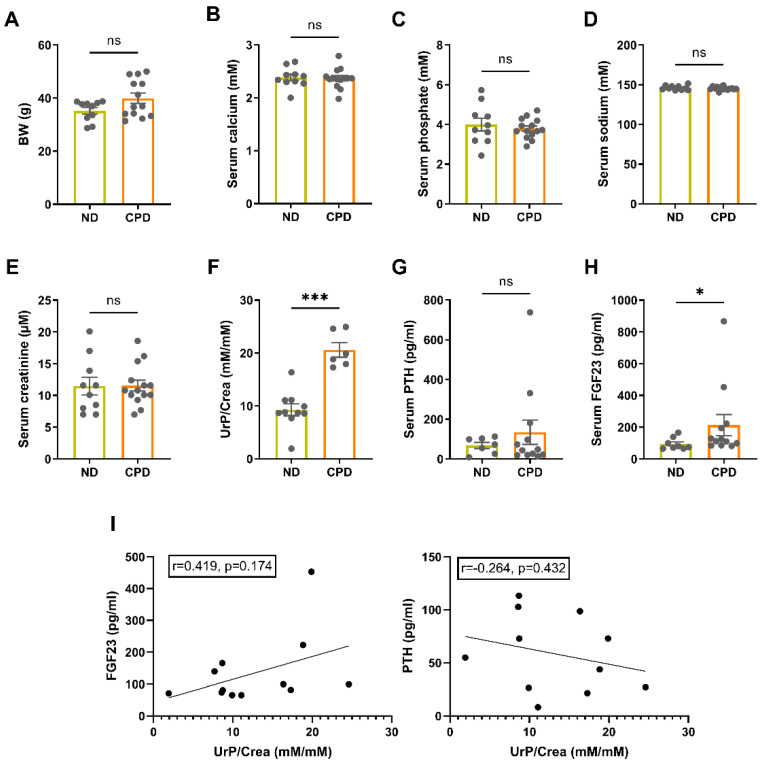
Mice maintained on CPD for 14 months have increased levels of FGF23, but normal kidney function. (**A**) Mice fed the CPD have comparable body weight (BW) to animals fed the ND (*n* = 10–13). (**B**–**E**) CPD mice are normocalcemic, normophosphatemic, and normonatremic (*n* = 10–14). (**E**) Serum creatinine levels are unchanged between CPD and ND mice (*n* = 10–14). (**F**) Urinary phosphate excretion relative to creatinine (UrP/Crea) is significantly increased in CPD mice compared to ND controls (*n* = 6–10). (**G**) Serum PTH levels are not changed between ND and CPD mice (*n* = 7–12). (**H**) CPD mice present with increased concentrations of circulating intact FGF23 (*n* = 8–12). (**I**) Correlation analysis of serum FGF23 and PTH with urinary phosphate excretion (*n* = 11–12). Bars in (**A**–**H**) represent mean ± SEM for ND and CPD mice. * *p* < 0.05, *** *p* < 0.001 by Mann–Whitney U-test. Insets show Pearson correlation coefficients. ns, not significant.

To establish a model of long-term excessive dietary phosphate intake not associated with secondary hyperparathyroidism, we fed male WT mice a calcium, phosphate, and lactose enriched diet (CPD) after weaning for 14 months and compared them with age-matched controls fed a normal mouse diet (ND). Both diets had a similar Ca/P ratio (1.43 for ND and 1.60 for CPD), the same energy content, and the same distribution of major nutrients (carbohydrates, protein, fat). The high lactose content of the CPD is used to facilitate paracellular, vitamin D independent, absorption of calcium and phosphate in the gut [26]. Mice fed the CPD had comparable body weight (BW), relative to animals fed the ND (Figure 1A). Furthermore, mice on the CPD were normocalcemic, normophosphatemic, normonatremic, and showed unchanged serum levels of creatinine, suggesting normal kidney function (Figure 1B–E). Urinary phosphate excretion was significantly increased in the CPD mice compared to ND controls (Figure 1F). As expected, mice kept on CPD did not present with elevated levels of intact PTH (Figure 1G), but showed increased concentrations of circulating intact FGF23 (Figure 1H). In accordance with the notion that the rise in circulating intact FGF23 occurred as a compensatory mechanism to enhance urinary phosphate excretion in response to an increase in dietary phosphate intake, we found a positive, albeit statistically non-significant, correlation between FGF23 and urinary phosphate excretion (Figure 1I). The correlation between PTH and urinary phosphate excretion was much weaker and actually negative (Figure 1I). These findings support the idea that in our model of dietary phosphate excess separates between the two phosphaturic hormones, resulting in a selective upregulation of FGF23, because a rise in PTH is largely prevented by the normal Ca/P ratio of the CPD.

Hence, we successfully established a mouse model of long-term excessive dietary phosphate intake, characterized by normophosphatemia, normocalcemia, elevated intact FGF23, but largely normal PTH.

### 3.2. High Phosphate Intake in Aged Mice Leads to Hypertension, but Not Left Ventricular Hypertrophy

**Figure 2:**
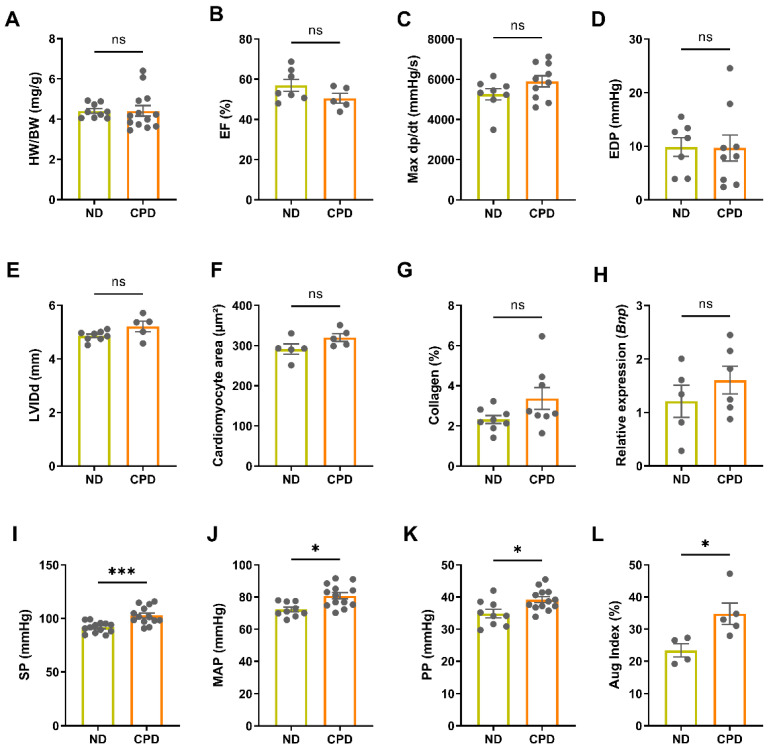
High phosphate intake in aged mice leads to hypertension, but not to left ventricular hypertrophy. (**A**) Heart weight-to-body weight ratio (HW/BW) is not increased in mice on CPD, relative to ND controls (*n* = 10–13). (**B**–**E**) Ejection fraction (EF), MaxdP/dt, end-diastolic pressure (EDP), and LV internal diameter (LVID) are unchanged between CPD and ND mice (*n* = 5–10). (**F**) Cardiomyocyte size is unchanged in CPD and ND mice (*n* = 5). Collagen content (**G**) and cardiac mRNA expression of *Bnp* (**H**) is similar between ND and CPD mice (*n* = 5–9). (**I**–**K**) Systolic (SP), mean arterial (MAP), and pulse pressure (PP) are significantly increased in mice fed CPD, relative to ND (*n* = 11–15). (**L**) CPD mice have increased arterial stiffness as shown by an increase in the augmentation index (Aug Index) (*n* = 4–5). Bars in (**A**–**L**) represent mean ± SEM for ND and CPD mice. * *p* < 0.05, *** *p* < 0.001 by Mann–Whitney U-test. ns, not significant.

Several earlier studies have reported LV hypertrophy in mice fed a high phosphate diet [27,28]. To assess cardiovascular function in our model of long-term dietary phosphate excess, we phenotyped the mice via echocardiography, intraarterial and cardiac catheterization, heart histology, and expression analysis of cardiac hypertrophy markers. Heart weight-to-body weight ratio was not increased in mice on CPD, relative to ND controls (Figure 2A). Moreover, left ventricular function was unaltered between the groups, as evidenced by unchanged ejection fraction (EF), MaxdP/dt, end-diastolic pressure (EDP), and LV internal diameter (LVID) (Figure 2B–E). Furthermore, mice fed the CPD diet did not present with LV hypertrophy as evidenced by unchanged cardiomyocyte area measured by wheat germ agglutinin (WGA) staining, unchanged LV collagen content measured by picrosirius red staining, and similar cardiac mRNA expression of the molecular marker of hypertrophy, *Bnp* (Figure 2F–H). Notably, cardiovascular phenotyping revealed a significant increase in systolic and mean arterial pressure as well as pulse pressure in mice fed CPD, relative to ND control mice (Figure 2I–K). To further elucidate the factors that may cause hypertension in mice on CPD, we measured augmentation index. The augmentation index is an indirect measure of arterial stiffness. Arterial stiffness increases with age [29], and is associated with elevations in systolic and diastolic blood pressure [30,31]. Pressure wave analysis revealed increased arterial stiffness as shown by an increase in the augmentation index in the CPD group, suggesting increased peripheral resistance in these mice (Figure 2L).

Together, these data demonstrate increased arterial blood pressure and increased arterial stiffness in aged mice on CPD, but strongly argue against LV hypertrophy and LV functional impairment in these mice.

### 3.3. Chronically Elevated Dietary Phosphate Intake Stimulates the Renin–Angiotensin–Aldosterone System and Increases Sympathetic Activity

**Figure 3:**
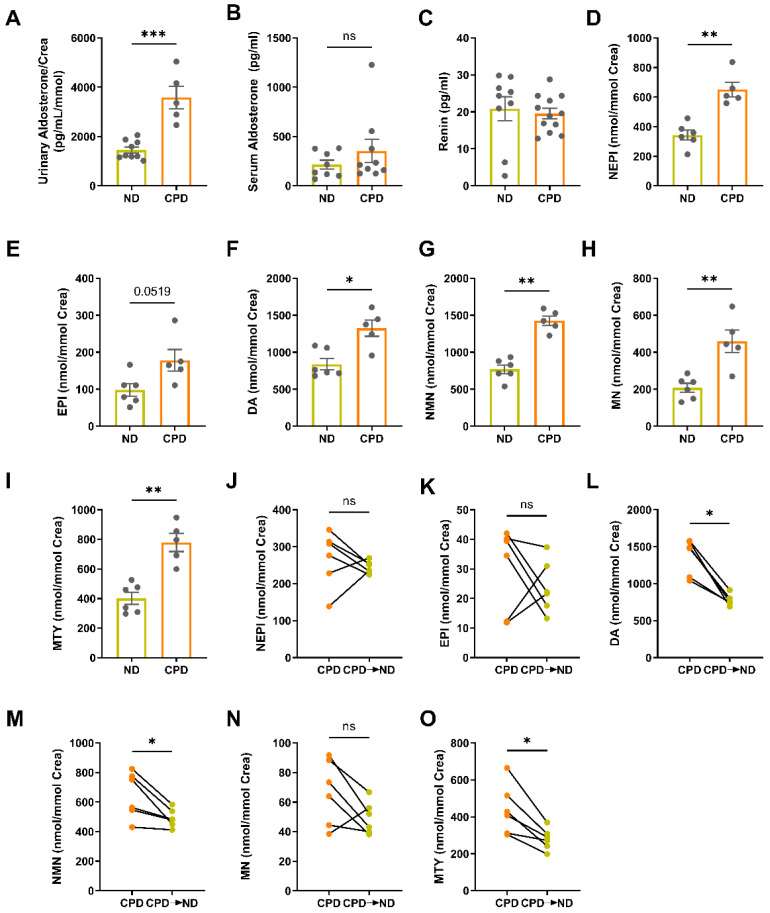
Chronically elevated dietary phosphate intake stimulates the renin–angiotensin–aldosterone system (RAAS) and increases sympathetic activity. (**A**) Urinary aldosterone secretion is increased in CPD mice (*n* = 5–9). (**B**) Serum aldosterone and (**C**) serum renin concentrations are unchanged between mice fed CPD and ND (*n* = 8–12). (**D**–**I**) Urinary excretion of the catecholamines norepinephrine (NEPI), dopamine (DA) and the catecholamine metabolites normetanephrine (NMN), metanephrine (MN) and methoxytyramine (MTY) are significantly elevated in mice fed the CPD compared to mice on ND, while epinephrine (EPI) did not reach statistical significance (*n* = 5–6). Levels of (**J**) norepinephrine, (**K**) epinephrine and (**N**) metanephrine are not altered after switching the mice from CPD to ND for two weeks. The diet switch lowered levels of (**L**) dopamine, (**M**) normetanephrine and (**O**) metoxytyramine (*n* = 6). Bars in (**A**–**I**) represent mean ± SEM for ND and CPD mice. * *p* < 0.05, ** *p* < 0.01, *** *p* < 0.001 by Mann–Whitney U-test or Wilcoxon matched-pairs test. ns, not significant.

Next, we examined two major regulators of arterial stiffness and vascular tone, the renin–angiotensin–aldosterone system (RAAS) and the sympathetic nervous system in our model of dietary phosphate excess. We reported previously that CKD mice fed the CPD have higher aldosterone levels compared with CKD mice on ND [32]. Besides the fact that the RAAS is a known regulator of blood pressure and vascular tone, it has been suggested that the RAAS is also an important determinant of arterial stiffness [33]. Notably, we found that urinary aldosterone secretion was increased in mice on CPD (Figure 3A). Serum aldosterone tended to be higher in mice on CPD, but this effect did not reach statistical significance (Figure 3B). Serum renin concentration did not show differences between mice on CPD and ND (Figure 3C).

The sympathetic nervous system is another known modulator of arterial stiffness, and has been shown to interact with the RAAS in individuals with hypertension [34,35,36]. To test whether mice on CPD have increased sympathetic tone, we measured urinary catecholamines and their metabolites, using mass spectrometry in 12–14 month-old mice [25]. Interestingly, norepinephrine (NEPI), dopamine (DA), metanephrine (MN), normetanephrine (NMN), and methoxytyramine (MTY) were all significantly elevated in mice fed the CPD, relative to the control mice on ND (Figure 3D–I). Epinephrine (EPI) tended to be higher in CPD mice, but this effect did not reach statistical significance (Figure 3E). To determine if the observed effect was indeed caused by CPD and if it was reversible, we switched aged mice maintained on CPD since weaning to ND for two weeks, and subsequently repeated the measurements. In most mice, the urinary concentration of catecholamines and catecholamine metabolites decreased after switching them from CPD to ND (Figure 3J–O). However, this effect reached statistical significance only for dopamine (DA), normetanephrine (NMN), and methoxytyramine (MTY) (Figure 3L,M,O).

In conclusion, our data show that excess dietary phosphate induces increased blood pressure and increased arterial stiffness, and that these effects are accompanied by an activation of the RAAS and a stimulation of the sympathetic tone.

## 4. Discussion

The main goal of this study was to evaluate for the first time the effects of long-term excess dietary phosphate intake on the cardiovascular system in aged, healthy mice, independent of secondary hyperparathyroidism. Here, we show that chronic exposure to high dietary phosphate in 14-month-old mice caused increased blood pressure and increased arterial stiffness despite normophosphatemia, and that these changes were associated with an activation of the RAAS and a stimulation of the sympathetic tone.

Male C57BL/6 mice maintained for 14 months on CPD in our study demonstrated an increase in systolic, mean arterial, and pulse pressure, independent of hyperphosphatemia or increased intact PTH. Furthermore, the mice on CPD were characterized by enhanced arterial stiffness as evidenced by an increase in augmentation index. Increased arterial stiffness is a precursor for hypertension [37,38]. In line with our findings, the recent intervention study by Mohammad and coworkers [10] reported a small increase in arterial blood pressure in healthy individuals in response to a high phosphate diet. However, the latter investigators failed to detect changes in arterial elasticity induced by dietary phosphate loading [10]. Previous studies examining the acute effects of a phosphate load in non-CKD patients also failed to observe changes in augmentation index or pulse wave velocity [39]. The reason for these discrepancies is currently unclear. It is conceivable that arterial stiffening induced by excessive dietary phosphate in healthy subjects is only seen after long-term exposure to increased dietary phosphate.

To further decipher the pathophysiological process(es) by which phosphate causes an increase in blood pressure, we measured aldosterone levels in serum and urine as well as serum renin concentration, and assessed urinary secretion of catecholamines. We found urinary aldosterone/creatinine concentrations to be significantly elevated in mice fed the CPD. This finding suggests that activation of RAAS may at least partially be responsible for the increased arterial blood pressure in the CPD group. Interestingly, serum renin concentration remained unchanged in mice on CPD, suggesting that the CPD-induced activation of RAAS occurs downstream of renin secretion. In addition, we found an increase in urinary concentration of catecholamines and their metabolites in mice fed the high phosphate diet, suggesting sympathoadrenergic activation. Similarly, healthy subjects placed on a high phosphate diet showed an increase in urinary metanephrine and normetanephrine excretion [10]. Catecholamines are endogenous neurotransmitters and hormones derived from tyrosine metabolism, and are essential for maintenance of metabolic and cardiovascular homeostasis and for adaptation to stressors [40,41]. The most abundant catecholamines in circulation are norepinephrine, epinephrine, and dopamine [42]. Catecholamines have many cardiovascular and metabolic actions, including increasing the heart rate, blood pressure, myocardial contractility, and cardiac conduction velocity. The *O*-methylated metabolites of norepinephrine, epinephrine and dopamine are normetanephrine (NMN), metanephrine (MN), and methoxytyramine (MTY), respectively [41]. Metanephrine and normetanephrine are referred to as metanephrines. It has been proposed that metanephrines can be used as markers of sympathetic activity [43]. Indeed, two independent studies have found a strong association between urinary metanephrine levels and systolic blood pressure in humans [41,44]. Therefore, the partially reversible increase in urinary excretion of catecholamines induced by the CPD in our study may contribute to increased arterial blood pressure and arterial stiffness, in addition to RAAS activation.

It is clear that the key question is the mechanism for how dietary phosphate may influence cardiovascular function. The effect of phosphate on the cardiovascular system may be direct, or mediated indirectly through one of the hormones involved in its homeostasis. Our study suggests that the untoward cardiovascular effects of a high dietary phosphate intake are independent of PTH. It is of course tempting to speculate that the increase in circulating FGF23 induced by excessive phosphate intake in CPD mice upregulated RAAS activity and sympathetic tone, thereby causing elevated blood pressure and increased arterial stiffness. As a matter of fact, there is accumulating evidence suggesting that FGF23 and the RAAS may interact [45]. In this context, it has been shown in mice that FGF23 suppresses the renal angiotensin converting enzyme-2 (ACE2), shifting the balance between the vasodilatory angiotensin (1–7) and the ACE1-produced angiotensin-2 towards the vasoconstrictive and prohypertensive angiotensin-2 [46,47]. Hence, FGF23 may directly activate the classical, prohypertensive RAAS by increasing angiotensin-2 production, which may subsequently induce a rise in peripheral resistance and aldosterone secretion. In line with this notion, we found unchanged renin concentrations in CPD mice, relative to mice kept on ND. However, it is unclear whether this is also true for humans because a recent study in hemodialysis patients with LVH failed to find an association between FGF23 levels and components of the RAAS [48].

Based on our data and the study by Mohammad and coworkers in healthy humans, high dietary phosphate intake stimulates sympathoadrenergic activity. However, the mechanistic link between FGF23 signaling and sympathoadrenergic activity has yet to be defined. It is interesting to note in this context that FGF23 has been shown to stimulate rostral ventrolateral medulla presympathetic neuron activity in the brainstem [49]. Conversely, it is also possible that sympathetic activation might be responsible for the changes in FGF23 serum levels in mice fed the CPD. In the latter context, Kawai and coworkers reported that acute dosing with the beta-adrenergic agonist isoproterenol increased *Fgf23* transcription in bone, and that this effect was reverted after administration of the beta blocker propranolol [50]. On the other hand, normalization of PTH and FGF23 in healthy adults on a high phosphate diet did not normalize blood pressure in these individuals, they rather remained hypertensinogenic, suggesting that the effects of phosphate are not mediated by FGF23 or PTH. Thus, the question of how dietary phosphate influences cardiovascular function remains controversial.

In conclusion, here we present a novel mouse model of long-term dietary phosphate excess characterized by increased blood pressure and arterial stiffness, normophosphatemia, normal PTH, but elevated FGF23. Our model may be useful to disentangle the complex relationship between phosphate, calcium-regulating hormones, FGF23, RAAS, sympathoadrenergic activity, and the cardiovascular system in health and disease.

## Data Availability

All data generated or analyzed in this study are included in this article.
